# Development and Characterization of Near-Isogenic Lines Revealing Candidate Genes for a Major 7AL QTL Responsible for Heat Tolerance in Wheat

**DOI:** 10.3389/fpls.2020.01316

**Published:** 2020-08-28

**Authors:** Lu Lu, Hui Liu, Yu Wu, Guijun Yan

**Affiliations:** ^1^Faculty of Science, UWA School of Agriculture and Environment, The University of Western Australia, Perth, WA, Australia; ^2^The UWA Institute of Agriculture, The University of Western Australia, Perth, WA, Australia; ^3^Chengdu Institute of Biology, Chinese Academy of Sciences, Chengdu, China

**Keywords:** heat tolerance, near-isogenic lines, 7AL, quantitative trait loci, single nucleotide polymorphism assay, candidate genes, wheat

## Abstract

Wheat is one of the most important food crops in the world, but as a cool-season crop, it is more prone to heat stress, which severely affects crop production and grain quality. Heat tolerance in wheat is a quantitative trait, and the genes underlying reported quantitative trait loci (QTL) have rarely been identified. Near-isogenic lines (NILs) with a common genetic background but differing at a particular locus could turn quantitative traits into a Mendelian factor; therefore, they are suitable material for identifying candidate genes for targeted locus/loci. In this study, we developed and characterized NILs from two populations Cascades × Tevere and Cascades × W156 targeting a major 7AL QTL responsible for heat tolerance. Molecular marker screening and phenotyping for SPAD chlorophyll content and grain-yield-related traits confirmed four pairs of wheat NILs that contrasted for heat-stress responses. Genotyping the NILs using a 90K Infinium iSelect SNP array revealed five single nucleotide polymorphism (SNP) markers within the QTL interval that were distinguishable between the isolines. Seven candidate genes linked to the SNPs were identified as related to heat tolerance, and involved in important processes and pathways in response to heat stress. The confirmed multiple pairs of NILs and identified candidate genes in this study are valuable resources and information for further fine-mapping to clone major genes for heat tolerance.

## Introduction

Crop growth and productivity are often limited by abiotic stresses, especially heat and drought (Priya et al., 2019). Wheat is one of the most important food grain crops in the world, but being a cool-season crop, it often experiences heat stress. Each 1°C rise in temperature above the optimum can cause a 3–5% reduction in single grain weight under controlled environments (Dawson and Wardlaw, 1989) or ﬁeld conditions (Wiegand and Cuellar, 1981). Phenology is known to confound crop responses to heat; therefore, the effect of heat stress depends on its timing, duration, and frequency (Rezaei et al., 2015; Balla et al., 2019). While conventional breeding has developed some heat-tolerant lines, it is a time-consuming process, and the genetic and physiological basis of the improvements remain unclear (Driedonks et al., 2016). Understanding the underlying mechanism of heat tolerance and identifying candidate genes will help to accelerate the breeding of heat-stress-resilient genotypes (Budak et al., 2015).

Heat tolerance is a quantitative trait (Moffatt et al., 1990a; Yang et al., 2002) that involves complex genetic, physiological, and biochemical controls and is affected by environmental factors. Numerous heat tolerance QTL have been identified; for example, Yang et al. (2002) found QTL on the short arms of chromosomes 1B and 5A linked to grain filling duration; Mason et al. (2010, 2011) reported several QTL for heat susceptibility indices and yield traits on chromosomes 1A, 1B, 2A, 2B, 3B, 5A, and 6D; Paliwal et al. (2012) reported QTL on chromosomes 2B, 7B, and 7D for thousand-grain weight, grain fill duration, and canopy temperature depression, respectively; Vijayalakshmi et al. (2010) reported QTL on chromosomes 2A, 3A, 4A, 6A, 6B, and 7A with significant effects on grain yield, grain weight, grain filling, stay green, and senescence-associated traits under post-anthesis high-temperature stress in wheat. Most of these reported QTL have been based on mapping using low-density simple sequence repeat (SSR) markers and/or amplified fragment length polymorphism (AFLP) markers. Talukder et al. (2014) increased the marker density to 972 molecular markers and identified QTL associated with different traits related to heat tolerance in wheat. They found that QTL QHtscc.ksu-7A on chromosome 7A was consistently identified for traits thylakoid membrane damage (TMD), plasma membrane damage (PMD), and SPAD chlorophyll content (SCC), with high logarithm of odds (LOD) values ranging from 4.15 to 6.95 and explaining high phenotypic variations ranging from 18.9 to 33.5%. This major QTL QHtscc.ksu-7A, with flanking markers Xbarc121 and Xbarc49, was chosen as the target locus for developing NILs in this study.

It remains challenging to use QTL markers directly in breeding programs due to the large genomic intervals of the most identified QTL (Mia et al., 2019). One solution for identifying candidate genes and closely linked markers is to develop NILs, which turn quantitative traits into Mendelian factors (Liu et al., 2006). NILs are pairs of lines that have the same genetic background between isolines, except for the targeted locus (Dorweiler et al., 1993). NILs make it easier to study phenotypic impacts attributable to a specific gene or locus (Pumphrey et al., 2007). Characterizing NILs through genotype–phenotype association analyses can lead to the identification of candidate genes (Mirdita et al., 2008; Liu et al., 2010; Mia et al., 2019; Wang et al., 2019). Traditionally, NILs development has been considered time-consuming and tedious (Tuinstra et al., 1997). By combining a fast generation cycling system (FGCS) (Zheng et al., 2013) with heterogeneous inbred family (HIF) method and repeated DNA marker-assisted selection (MAS) (Tuinstra et al., 1997), the NIL development process can be shortened to about six generations per year (Yan et al., 2017).

Single nucleotide polymorphism (SNP) markers are high-density DNA markers widely used in genetic studies, including genetic diversity, phylogenetic relationships, and marker-trait associations, such as genome-wide association study (GWAS) or QTL mapping (Wang et al., 2014; Cabral et al., 2018). The 90k SNP array, developed from hexaploid wheat and *Aegilops tauschii* sequences (Wang et al., 2014) with dense coverage of the wheat genome, has been extensively harnessed for genetic research (Cavanagh et al., 2013; Avni et al., 2014; Colasuonno et al., 2014; Russo et al., 2014; Sukumaran et al., 2015). Due to its efficiency of characterizing genetic resources and discriminating between closely related lines (Rimbert et al., 2018), 90k SNP genotyping was used in this study to characterize the developed NILs, in combination with phenotyping under controlled environments.

The objectives of this study were to 1) develop and confirm NILs targeting the major heat tolerance QTL on chromosome 7A, 2) identify candidate gene(s) underlying the 7A QTL responsible for heat tolerance by genotypic and phenotypic characterization of the NILs, and 3) shed light on the genetic mechanism of heat tolerance in wheat by inspecting this major genomic region and investigating its underlying candidate genes.

## Materials and Methods

### Plant Materials and Selection of Crossing Parents

In a previous study, 499 wheat genotypes from a variety of sources were screened and evaluated for heat-stress responses (Hameed, 2015). Among them, cultivars Tevere and W156 showed heat tolerance at both the seedling and reproductive stages with high yield, whereas Cascades (Aroona//Tadorna/Inia 66) was sensitive at both stages (unpublished data). Cascades and Tevere are two common wheat cultivars and W156 is a landrace, which originated from Australia, Italy, and India, respectively. When the flanking marker Xbarc49 of the targeted 7A QTL was used for genotyping the three cultivars, heat-tolerant Tevere and W156 showed the tolerance allele at 216 bp, and heat-susceptible Cascades showed the susceptibility allele at 203 bp. The three cultivars were therefore used to establish two cross populations, Cascades/W156 and Cascades/Tevere, for the development of NILs targeting the 7A locus.

### Development of NILs

**Figure 1:**
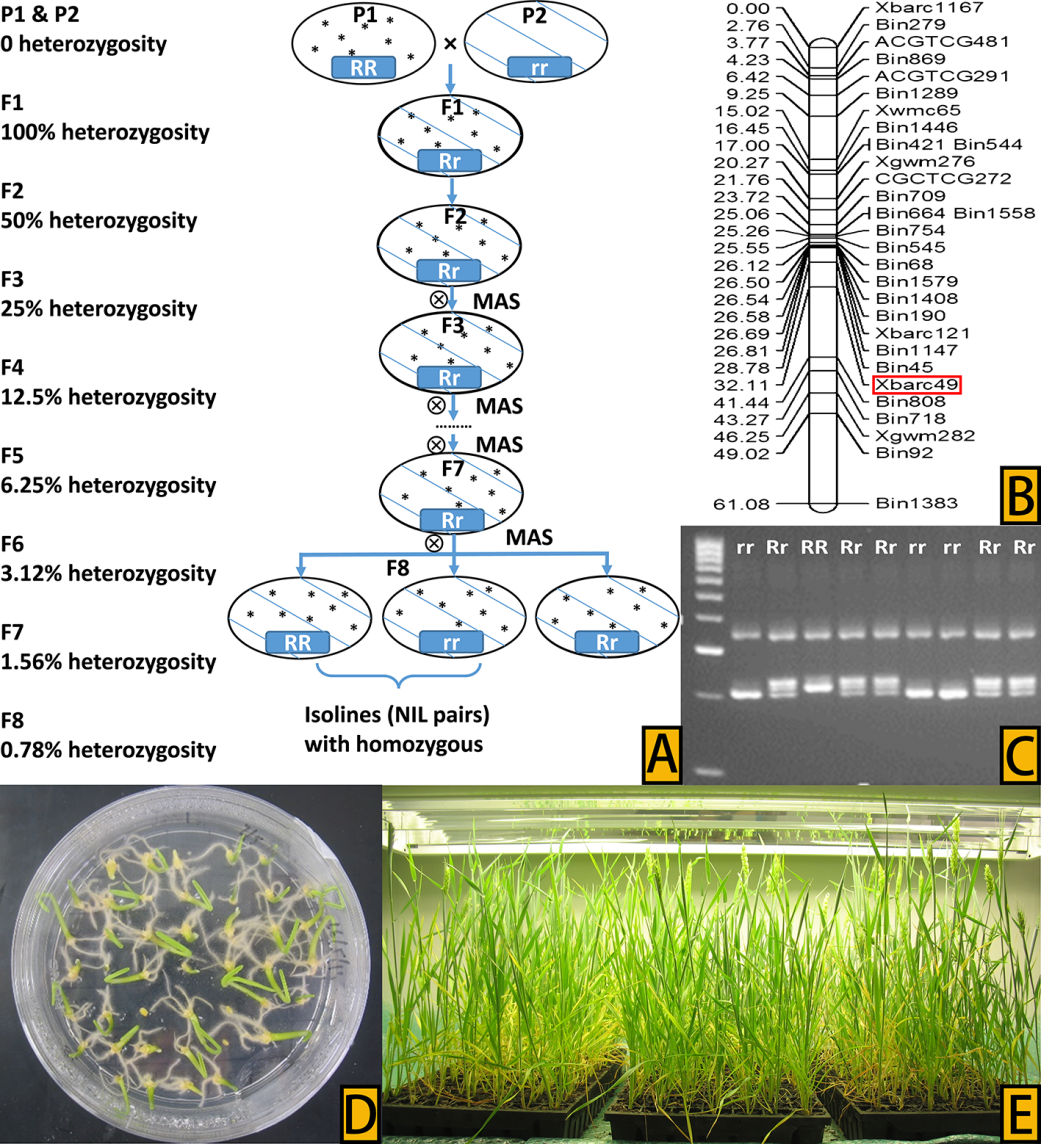
**(A)** Process of the HIF method to develop NIL pairs, with percentage heterozygosity in each generation shown on the left of the flow chart. **(B)** QTL hotspot in wheat chromosome 7A and the marker (in the box) used for selection, adapted from Talukder et al., 2014. **(C)** Marker-assisted selection of different progeny types, with tolerance progeny, susceptible progeny, and heterozygous progeny marked RR, rr, and Rr, respectively, on the top of the gel bands. **(D)** Culture of young embryos on Petri plates in a sterile medium. **(E)** Seedlings from embryo culture growing in the plant growth chamber.

NILs were developed from the two populations using the HIF method (Tuinstra et al., 1997) in combination with embryo-culture-based FGCS (Zheng et al., 2013; Yan et al., 2017), following a similar procedure as described in Wang et al. (2019). Specifically, MAS started from the second generation of progenies (F2) (**Figure 1A**) where genomic DNA was isolated from two-week-old seedlings of each plant using a modified CTAB method (Murray and Thompson, 1980). Xbarc49 (**Figure 1B**), the flanking marker of QHtscc.ksu-7A (Talukder et al., 2014), was used to identify heterozygous progenies (Rr) from the two cross populations (**Figure 1C**). PCR reactions were performed, and amplified products viewed following the protocol described in Wang et al. (2018). MAS of heterozygous progenies, together with embryo-culture-based FGCS (**Figures 1D, E**), continued until the eighth generation of progenies (F8); at F8, only those homozygous progenies from single seed descent with the tolerance allele (RR/+) from either W156 or Tevere and those with susceptibility allele (rr/–) from Cascades were selected as pairs of candidate NILs. Thirteen pairs of F8 NILs, numbered from NIL1 to NIL13, developed from the two populations were used as putative NIL pairs to screen their phenotypes for performance under heat stress.

### Plant Growth Conditions and Heat Treatments

The seeds of all isolines were germinated in water on Petri dishes, before sowing one plant per pot (8 cm × 8 cm × 16 cm) containing soil media (5:2:3 compost:peat:sand, pH 6.0) (Mia et al., 2019). For each isoline, six pots (three replicates each for the control and the heat-stress treatment) were grown in a naturally lit glasshouse at The University of Western Australia, Crawley, Western Australia (31°59’ S, 115°49’E). The plants were fertilized fortnightly with ‘Diamond Red’ (Campbells Fertilisers Australasia Pty Ltd, Australia) from four weeks after sowing until the end of the grain-filling period. The glasshouse environment (temperature, relative humidity, and light intensity) is detailed in **Supplementary Table S1**. The experiment was arranged in a completely randomized block design.

Anthesis date, as Zadoks’ growth scale Z60 (Zadoks, 1974), of each plant was recorded by tagging each plant on the wheat head where the first anther appeared. The time point to start the heat treatment, treatment temperature, and other settings were as per previous studies (Pradhan et al., 2012; Talukder et al., 2014; Shirdelmoghanloo et al., 2016a). Specifically, on the 10th day after anthesis (DAA), the three treatment pots were moved into a growth chamber set to 37/27°C (day/night), 14-h photoperiod, and 420 μmol m^−2^ s^−1^ light intensity for the 3-day heat treatment. Enough water was given to the plants to ensure there was no drought stress, only heat stress. The pots were returned to the glasshouse after the heat treatment.

### Phenotype Screening

Chlorophyll contents were measured on flag leaves using a handheld portable chlorophyll meter (SPAD-502Plus; Konica Minolta, Osaka) for both the control and heat-stressed plants. Time points for measurements followed the procedures described in Pradhan et al. (2012). Changes in SPAD chlorophyll contents (ΔSCC) in flag leaves were calculated based on differences in chlorophyll contents before (as ‘Chl10’ at 10 DAA) and after (as ‘Chl13’ at 13 DAA) treatment, as follows: ΔSCC = mean of Chl10 – mean of Chl13 (Shirdelmoghanloo et al., 2016b). Measurements and calculations were the same for controls.

Agronomic traits (grain number and grain yield per plant) were measured after harvest for plants in the control and heat-stressed treatments. The performance differences in final yield between isoline pairs were determined by subtracting mean values in the control from those in the heat treatment.

Statistical analyses were undertaken using t-tests to compare phenotypic variation in the NIL pairs. True NILs were confirmed if significant differences existed in the performance of tested traits between isolines, and their resistant and susceptible phenotypes matched their genotypes of Xbarc49’s RR/+ and rr/ – alleles, respectively.

### Genotyping by 90k Infinium iSelect SNP Array

The 90K SNP array was used for genotyping, with genotype–phenotype associations used to identify candidate gene(s) (Alaux et al., 2018). Specifically, genomic DNA samples of the confirmed NILs were genotyped using the Wheat 90K Illumina iSelect Array (Wang et al., 2014). SNP clustering and genotype calling were performed using GenomeStudio 2.0 software (Illumina). SNPs with a call frequency <0.8 (i.e., missing data points >20%), minor allele frequency (MAF) <0.05 or heterozygous calls >0.25 were removed. SNP sequences that differed between NIL pairs were used to perform a BLAST search against the wheat reference genome (IWGSC, 2018). SNPs located on the 7AL chromosome arm, especially those within the marker interval of QHtscc.ksu-7A (Talukder et al., 2014), were scrutinized using JBrowse (http://www.wheatgenome.org/Tools-and-Resources/Sequences) for candidate gene discovery.

## Results

### Four NIL Pairs Confirmed With Significant Differences in Chlorophyll Content

After comparing the ΔSCC data for NIL pairs, no significant differences were observed among the 13 putative NIL pairs grown in the non-stressed (control) treatment, whereas the isolines of four NIL pairs significantly differed in the heat-stressed treatment. Of these, the isolines with positive alleles (+NILs) had smaller reductions in SPAD chlorophyll content than isolines with negative alleles (–NILs). The isolines of NILs 5, 10, and 13 differed significantly at P < 0.05, and NIL 9 differed significantly at P < 0.01 (**Table 1**).

**Table 1:** Reduction of SPAD chlorophyll content (ΔSCC) in the confirmed NIL pairs.

NIL name	Population	ΔSCC (Chl10–Chl13)			
		Control		Treatment	
		Mean	P-value	Mean	P-value
NIL5(+)	Cascade/Tevere	–0.4	ns	0.53	*
NIL5(–)		–0.8		7.13	
NIL9(+)	Cascade/W156	1.1	ns	4.57	**
NIL9(–)		5.05		7.97	
NIL10(+)	Cascade/W156	0.67	ns	1.7	*
NIL10(–)		0.6		12.23	
NIL13(+)	Cascade/Tevere	0.15	ns	1.7	*
NIL13 (–)		1.27		6.7	

ns = non-significant at P ≤ 0.05; * = significant at P ≤ 0.05; ** = significant at P ≤ 0.01. The statistics was done using t-test. (+) indicates isolines with resistance allele from W156 or Tevere; (–) indicates isolines with susceptibility allele from Cascades.

### Tolerant Isolines Performed Better Than Susceptible Isolines on Other Agronomic Traits in the Confirmed NlLs

**Figure 2:**
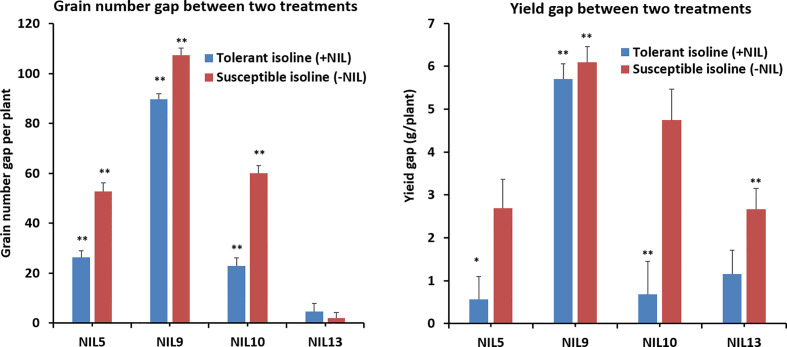
Differences in agronomic trait gaps between the confirmed isolines of tolerant and susceptible alleles. Three replicates each for the control (naturally lit glasshouse with conditions described in detail in **Table S1**) and the heat-stressed treatment (growth chamber set at 37/27°C day/night) were used for statistical analysis. The column indicates trait gaps between the treatment and control, with error bar on top. * indicates significant difference at P ≤ 0.05; ** indicates significant difference at P ≤ 0.01. Statistics done using t-test.

Further investigation showed that significant differences existed between most of the treated isolines and their corresponding controls for yield-related traits. The differences or gaps in grain number per pot and yield per pot between the control and heat-stressed treatments are shown in **Figure 2** to compare yield performance in tolerant and susceptible isolines. For grain number, three of the four +NILs (5, 9, and 10) had smaller gaps than –NILs. For yield, all four +NILs had smaller gaps between non-stressed and stressed treatments than their counterparts. Generally, heat stress had smaller negative effects on grain number and yield in +NILs than –NILs.

### Five SNP Markers With Consistent Contrasting Genotypes in the Confirmed NIL Pairs

Of the 81,587 SNPs on the array, 53,052 were analyzed across the 21 chromosomes after removing those that did not meet the selection criteria. Analyzing the SNPs among the four confirmed pairs of isolines identified five SNPs within the 7AL QTL region with consistent contrasting genotypes between the resistant and susceptible isolines (**Table 2**).

**Table 2:** SNPs showing consistent contrast callings in the confirmed four pairs of NILs.

No.	Marker Name	SNP	Physical location
1	BS00071558_51	[T/C]	chr7A:626897156.626897256
2	wsnp_Ku_c5160_9203226	[T/C]	chr7A:626897816.626898016
3	wsnp_Ku_rep_c113718_96236830	[A/G]	chr7A:625739519.625739719
4	wsnp_Ra_c26491_36054023	[A/G]	chr7A:621582917.621583094
5	wsnp_RFL_Contig2864_2688208	[T/C]	chr7A:625640069.625640169

### Candidate Genes Identified by Blasting the Wheat Reference Genome

Seven candidate genes were identified within the QHtscc.ksu-7A region by blasting the above five SNP markers with wheat reference genome RefV1.0. The annotations for high and low confidence genes of RefV1.0 were used, with various databases compared, including NCBI (https://www.ncbi.nlm.nih.gov) and InterMine (http://www.wheatgenome.org/Tools-and-Resources/Sequences), to determine possible gene functions (**Table 3**).

**Table 3:** Function annotations of candidate genes.

Gene	Physical position	Database	Identifier	Description
*TraesCS7A01G612600LC*	626894752.626898021	EMBL-EBI	BQ245642 HX055146 HX055177 CJ956871 CJ945027	Yield improvement under stress response to blast fungus wheat responses to fungi infections response to powdery mildew infection responses to fungi infections
*TraesCS7A01G432000*	625737761.625740656	Interpros	IPR013083	Zinc finger, RING/FYVE/PHD-type
*TraesCS7A01G431600*	625635655.625640991	GOs Interpros Pfams	GO:0005515 IPR001810 IPR015915 IPR000014 IPR011498 PF13426 PF13415 PF07646 PF13418 PF12937	Molecular Function: protein binding F-box domain Kelch-type beta propeller PAS domain Kelch-repeat type 2 PAS domain Galactose oxidase, central domain Kelch motif Galactose oxidase, central domain F-box-like
*TraesCS7A01G428200*	621564527.621567165	GOs Interpros Pfams	GO:0020037 GO:0055114 GO:0004601 GO:0006979 IPR000823 IPR002016 IPR010255 IPR019793 IPR019794 PF00141	MF: heme binding BP: oxidation-reduction process MF: peroxidase activity BP: response to oxidative stress Plant peroxidase Haem peroxidase, plant/fungal/bacterial Haem peroxidase Peroxidases heam-ligand binding site Peroxidase, active site Peroxidase
*TraesCS7A01G428400*	621856684.621857860			Similar function as gene *TraesCS7A01G428200*
*TraesCS7A01G430500*	624959655.624961254	GOs Interpros Pfams	GO:0016021 GO:0016020 GO:0055085 GO:0005215 GO:0022857 GO:0022891 IPR020846 IPR003663 IPR005828 IPR005829 PF00083	CC: integral component of membrane CC: membrane BP: transmembrane transport MF: transporter activity MF: transmembrane transporter activity MF: substrate-specific transmembrane transporter activity Major facilitator superfamily domain Sugar/inositol transporter Major facilitator, sugar transporter-like Sugar transporter, conserved site Sugar (and other) transporter
*TraesCS7A01G430600*	624965100.624967076	Interpros Pfams	IPR013126 IPR029047 IPR029048 IPR018181 PF00012	Heat shock protein 70 family Heat shock protein 70kD, peptide-binding domain Heat shock protein 70kD, C-terminal domain Heat shock protein 70, conserved site Hsp70 protein

**Figure 3:**
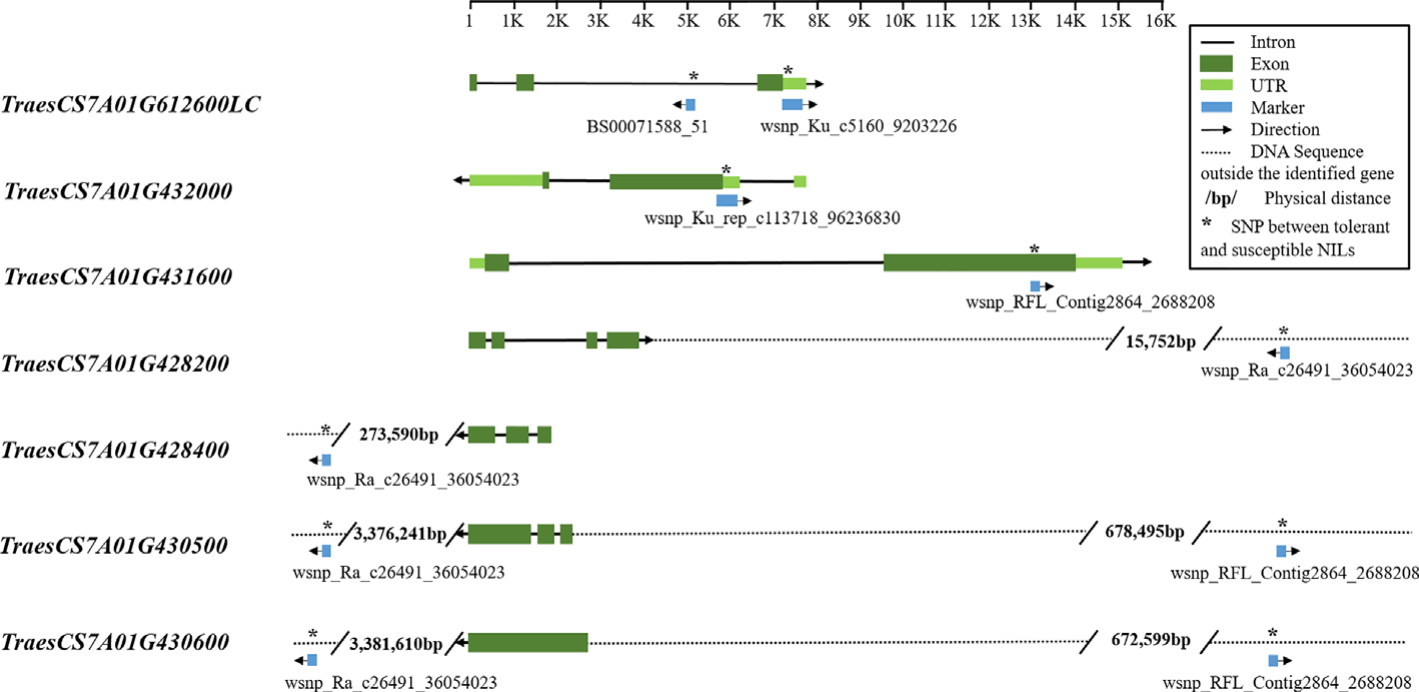
Candidate gene structures and SNPs between tolerant and susceptible NILs. The structural information of genes and SNP markers was extracted from the wheat genome database (https://urgi.versailles.inra.fr/jbrowseiwgsc/gmod_jbrowse). The SNP positions between tolerant and susceptible NILs are marked with an asterisk. For the first three genes, SNPs distinguishing tolerant and susceptible NILs were within the identified genes, either exon, intron, or untranslated region (UTR); for the other four genes, SNPs were outside the candidate genes and their physical distances to the genes are labeled.

Markers BS00071558_51 and wsnp_Ku_c5160_9203226 were co-located on gene *TraesCS7A01G612600LC*, with their SNP variations in the intron and untranslated region (UTR), respectively (**Figure 3**). Markers wsnp_Ku_rep_c113718_96236830 and wsnp_RFL_Contig2864_2688208 were co-located on gene *TraesCS7A01G432000* and *TraesCS7A01G431600*, respectively, with their SNP variations in the UTR and exon regions. Although no gene was co-located on wsnp_Ra_c26491_36054023, the marker was located close to genes *TraesCS7A01G428200* (15,752 bp away) and *TraesCS7A01G428400* (273,590 bp away), both of which function as peroxidase.

Blasting the sequence of *TraesCS7A01G612600LC* in NCBI identified five ESTs related to plant biotic or abiotic stress responses (Manickavelu et al., 2012). Genes *TraesCS7A01G432000* and *TraesCS7A01G431600* were involved in important biochemistry or molecular functions, such as protein binding, Zinc finger, F-box domain, Kelch motif, and Per-Arnt-Sim (PAS) domain, all of which are involved in various pathways of plant responses and reactions to external stimulus (Craig and Tyers, 1999; Adams et al., 2000; Prag and Adams, 2003; Hefti et al., 2004; van den Burg et al., 2008; Hennig et al., 2009; Moglich et al., 2009; Peng et al., 2012; Liu et al., 2015).

Apart from these genes, two other noteworthy genes—*TraesCS7A01G430500*, which functions as a sugar transporter family protein, and *TraesCS7A01G430600*, which functions as a heat shock protein (HSP)—were located in the interval between marker wsnp_Ra_c26491_36054023 and wsnp_RFL_Contig2864_2688208.

## Discussion

In this study, we reported the development of 13 putative NIL pairs targeting a major locus for heat tolerance. Among them, four pairs were confirmed as true NILs by genotype–phenotype association analysis. The confirmed NILs showed differential responses under non-stressed and heat-stressed conditions. NILs with alleles from the heat-tolerant parents (Tevere and W156) performed better than their counterparts in terms of physiological and agronomical traits, such as chlorophyll content, grain number, and grain yield. Characterization of these NIL pairs revealed that the presence of the tolerance allele significantly increased heat tolerance in the plants. One reason for the higher grain yield and grain number in the heat-tolerant NILs may be the positive correlation between chlorophyll content and gas exchange parameters reported in several studies (Chen et al., 2016; Wang et al., 2016).

Many physiological traits have been closely associated with heat response, such as canopy temperature, leaf senescence, night respiration, chlorophyll fluorescence, and cell membrane thermo-stability (Narayanan, 2018). The original QTL targeted in this study was associated with multiple traits, including thylakoid membrane damage (TMD), plasma membrane damage (PMD), and SPAD chlorophyll content (SCC) (Talukder et al., 2014). Membrane thermostability has a strong genetic correlation with grain yield in wheat (Reynolds et al., 1994; Fokar et al., 1998). Loss of chlorophyll content during grain filling has been associated with reduced yield under field conditions (Reynolds et al., 1994). TMD and PMD have also been associated with grain yield (Moffatt et al., 1990b; Saadall et al., 1990; Reynolds et al., 1994; Araus et al., 1998; Marcum, 1998; Blum et al., 2001; Wahid and Shabbir, 2005). Strong correlations among these traits suggest that these traits are under pleiotropic genetic control.

Leaf chlorophyll content is a major indicator of the photosynthetic capability of plant tissues (Rao et al., 2001; Pietrini et al., 2017). Some studies that focused on yield and photosynthetic traits (Raven and Griffiths, 2015; Gaju et al., 2016; Merchuk-Ovnat et al., 2016) have shown that the photosynthetic function duration of leaves is closely correlated to grain yield in wheat. Furthermore, spectral characteristics measured by SPAD are a good indicator for evaluating crop responses to high temperature (Talukder et al., 2014; Tao et al., 2016). Due to its correlation with yield and other performance indicators under heat stress, chlorophyll content measured by SPAD could be used as an easy and reasonable morphological marker for assessing heat tolerance, especially at the initial mass screening stage. The smaller the reduction in SPAD chlorophyll content (ΔSCC), the more tolerant the plant should be to heat stress. SPAD meters have been used to estimate leaf chlorophyll content in research and agricultural practices because it is a quick, simple and non-destructive method (Novichonok et al., 2016; Padilla et al., 2018; Zhao et al., 2018; de Souza et al., 2019; Galanti et al., 2019; Zhang et al., 2019). Here, agronomic traits such as grain number and grain yield were also measured to further confirm the true NIL pairs, which are valuable material for further studies of post-anthesis heat tolerance in wheat.

Except for the report by Talukder et al. (2014), the major QTL QHtscc.ksu-7A targeted in this study has been identified as responsible for heat tolerance in other studies. Vijayalakshmi et al. (2010) reported a 7A QTL linked to marker Xbarc121 for heat-tolerance traits, including Fv/Fm and time to maximum rate of senescence. Talukder et al. (2014) revealed that several ESTs, located in the same wheat deletion bin as Xbarc49, were related to stress response in different studies; therefore, the authors proposed that the major QTL QHtscc.ksu-7A was a genomic region rich in genes related to stress response. Dixit et al. (2015) hypothesized that multiple genes underpinned large-effect QTL. In this study, seven candidate genes within the targeted major 7AL QTL were identified as responsible for heat tolerance post-anthesis. The gene *TraesCS7A01G612600LC* is a homologous gene to *TraesCS7A01G612600L*; blasting *TraesCS7A01G612600L* by Blastx (translated nucleotide to protein) on NCBI with criteria of percentage identity ≥50% and e-value of <1e-5 revealed its origin from *Triticum urartu*, and it has been up-regulated at 24 h osmotic stress in the ABA-dependent signaling pathway (Li et al., 2019). Therefore, the gene *TraesCS7A01G612600LC* identified in this study supposedly has a similar function as its homologous gene. Abscisic acid (ABA) is generally considered as a stress signaling hormone, and the expression of stress-responsive genes in plants is primarily regulated by ABA-dependent and ABA-independent pathways (Agarwal and Jha, 2010; Yoshida et al., 2014). The ABA-dependent pathway is central to osmotic-stress responses in plants (Li et al., 2019). Moreover, five expression sequence tag (EST) markers associated with *TraesCS7A01G612600LC* were related to plant biotic or abiotic stress responses. Among these EST markers, HX055146, HX055177, CJ956871, and CJ945027 were related to wheat responses to fungi infections, including Fusarium head blight and powdery mildew (Manickavelu et al., 2012), while BQ245642 encoded a polypeptide useful for yield improvement by improving plant growth and development under at least one stress condition (Kovalic et al., 2007).

The annotation of genes *TraesCS7A01G432000* and *TraesCS7A01G431600* revealed their involvement in some important biochemistry or molecular functions, such as protein binding, Zinc finger, F-box domain, Kelch motif, PAS domain, and galactose oxidase. F-box domain genes are related to plant resistance, while F-box proteins are associated with cellular functions, such as signal transduction and regulation of the cell cycle during plant vegetative and reproductive growth and development (Craig and Tyers, 1999). For example, F-box protein FOA1 plays a role in ABA signaling involved in seed germination (Peng et al., 2012), ACRE189/ACIF1 regulates cell defense and death when tomato and tobacco are attacked by pathogens (van den Burg et al., 2008), Kelch motifs and kelch-repeat β-propellers undergo a variety of binding interactions with other molecules (Adams et al., 2000; Prag and Adams, 2003), and a PAS domain acts as a molecular sensor (Hennig et al., 2009; Moglich et al., 2009; Liu et al., 2015) and has been deemed as the key structural motif involved in protein-protein interactions during physiological reactions (Hefti et al., 2004). In summary, all these biological structures are extensively involved in various pathways of plant responses and reactions to external stimuli such that we can deduce that genes *TraesCS7A01G432000* and *TraesCS7A01G431600* regulate heat tolerance by controlling protein structures and protein binding on the biological structures mentioned above to achieve signal transduction, which is the key part in the pathway of heat tolerance.

The remaining four genes *TraesCS7A01G428200*, *TraesCS7A01G428400*, *TraesCS7A01G430500*, and *TraesCS7A01G430600* have functions as peroxidase, peroxidase, sugar transporter family protein, and HSP, respectively. The sugar transporter family protein is related to yield as sugar transport is one of the most important processes for plant development and their responses to biotic and abiotic factors (Lalonde et al., 2004; Lemoine et al., 2013). Tolerance to heat stress is frequently associated with maintaining sugar content in source leaves (Vasseur et al., 2011; Zhou et al., 2017). For example, a heat-tolerant tomato (*Solanum lycopersicum*) genotype had significantly higher fructose and sucrose contents in mature leaves than a heat-sensitive genotype under heat stress (Zhou et al., 2017). Peroxidase is related to stress tolerance—peroxidase activities increased significantly in a heat-tolerant wheat genotype in response to heat treatment (Sairam et al., 1998). Exposure to heat stress often leads to the generation of destructive ROS (reactive oxygen species), but plants have antioxidant mechanisms to escape excessive ROS. Several studies have shown that peroxidase plays an important role in antioxidant mechanisms and ameliorates the effects of heat stress in wheat (Suzuki et al., 2011; Caverzan et al., 2016). HSPs play a pivotal role as chaperones in conferring biotic and abiotic stress tolerance (Baniwal et al., 2004). The expression of HSP genes induced by high temperature can preserve the stability and function of intracellular proteins and protect them from denaturation through protein folding. They enhance membrane stability and detoxify ROS by positively regulating the antioxidant enzyme system. Additionally, HSP genes use ROS as a signal to molecules to induce HSP production. HSP also enhances plant immunity by accumulating and stabilizing pathogenesis-related proteins under various biotic stresses (Haq et al., 2019). Genotypes generating HSPs can withstand heat stress as they protect proteins from heat-induced damage (Farooq et al., 2011).

The gene structure analysis (**Figure 3**) identified several SNPs distinguishing tolerant and susceptible NILs located within the candidate genes, in which one marker wsnp_RFL-Contig2864_2688208 falls in the exon region. These markers can be used as functional markers, because not only does the exon encodes protein functional units but also noncoding DNA including intron and UTR has played significant roles in many studies (Cobb et al., 2008; Khurana et al., 2013; Lu et al., 2015; Grünewald et al., 2015). SNPs outside the identified candidate genes can still be used in marker-assisted selection for genetic and breeding research. Aharon et al. (2016) explored how far SNPs can be from the affected genes using a pathway-based approach and found that affected genes were often up to 2 Mbps from the associated SNP and not necessarily the closest to the SNP.

**Figure 4:**
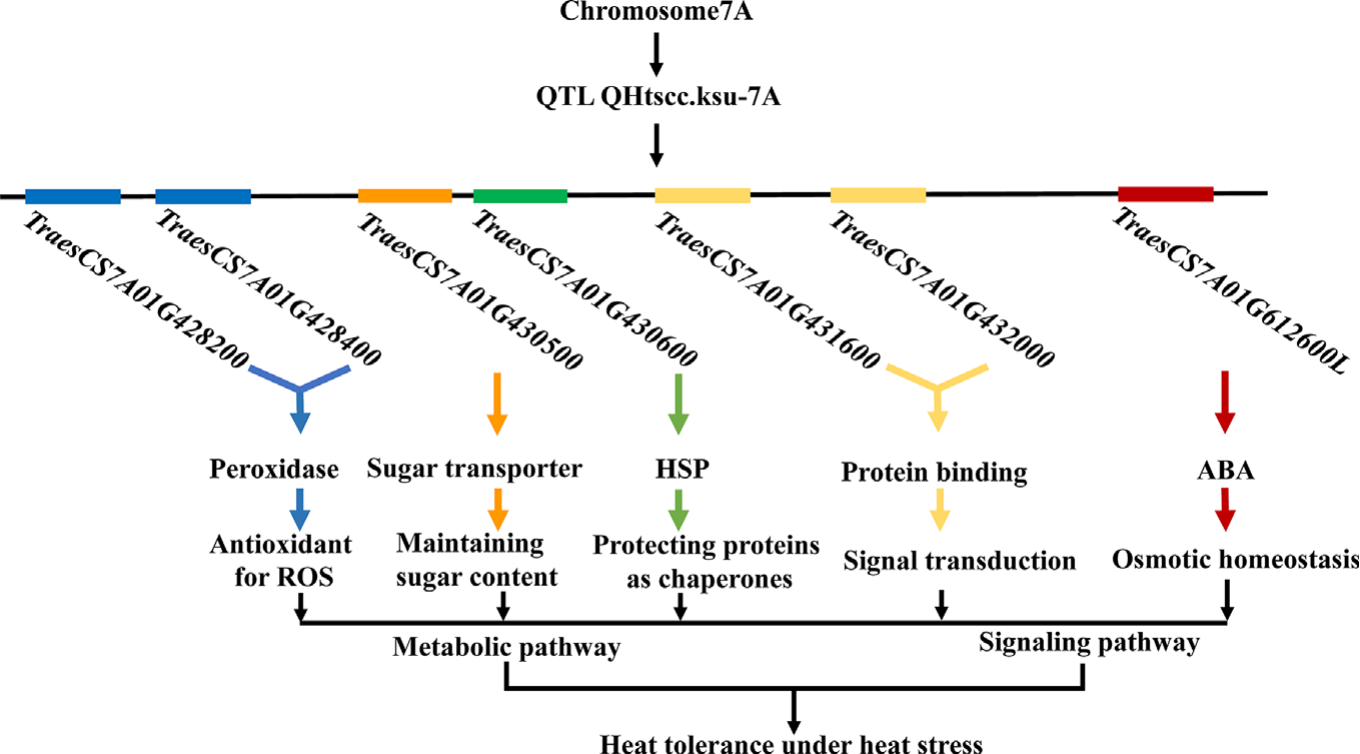
Postulated pathway based on the findings of this study showing a collaborative regulation network of multiple genes in wheat in response to heat stress. Signaling pathway and metabolic pathway involving a series of physiochemical processes and important molecules, including HSPs, antioxidants, metabolites, and hormones.

The molecular mechanisms underlying heat tolerance in wheat remain unclear. A schematic network, proposed to explain the mechanism of heat tolerance in legumes, suggested that signalling and metabolic pathways, involving a series of physiochemical processes and important molecules such as HSPs, antioxidants, metabolites, and hormones, play key roles in regulating the legume response to heat stress (Liu et al., 2019). The candidate genes identified in this study are consistent, to a large extent, with their proposed network. Therefore, we hypothesize that such a pathway might exist in wheat, where many key genes collaboratively regulate the crop’s response to heat stress (**Figure 4**).

## Conclusions

The NILs developed and validated in this study confirmed that the 7AL QTL, QHtscc.ksu-7A, is a major locus responsible for heat tolerance in wheat. The confirmed NILs and identified candidate genes are valuable resources for future studies in fine mapping and functional analyses of the chromosome region to clone the underlying gene(s).

## Data Availability Statement

All datasets presented in this study are included in the article/supplementary material.

## Author Contributions

LL, HL, and GY conceived and designed the study. HL developed NIL populations, and LL conducted the experiments in the plant growth chambers and greenhouse. LL collected the relevant data and performed the analysis with guidance from GY and HL. LL prepared the manuscripts with inputs from YW, HL, and GY. All authors contributed to the article and approved the submitted version.

## Funding

This research was funded by the Global Innovation Linkages Project (GIL53853) from the Australian Department of Industry, Innovation, and Science. The first author acknowledges the Research Training Program Scholarship from the Australian Government for sponsoring her Ph.D. study. The University of Western Australia provided funds for the open access publication fee.

## Conflict of Interest

The authors declare that the research was conducted in the absence of any commercial or financial relationships that could be construed as a potential conflict of interest.
